# A Self-Powered Vibration Sensing System for High-Voltage Transmission Lines with Equipotential Connections

**DOI:** 10.3390/s26113574

**Published:** 2026-06-04

**Authors:** Xueqiong Zhu, Jinggang Yang, Chengbo Hu, Zhen Wang, Ziquan Liu, Zhengyu Liu

**Affiliations:** State Grid Jiangsu Electric Power Company Limited Research Institute, Nanjing 211100, China

**Keywords:** transmission line monitoring, energy harvesting, graphene aerogel sensor, low-frequency vibration sensing, duty-cycled operation

## Abstract

In this work, a self-powered vibration sensing system is proposed, based on a spatial magnetic field energy harvester, a duty-cycled circuit module, a piezoresistive graphene-based vibration sensor, and a wireless communication unit. The energy harvester is capable of generating an output power of 729 μW under a magnetic field excitation of 0.11 mT at 50 Hz. The duty-cycled circuit module enables closed-loop self-powered operation of the sensing system by efficient power storage and periodic measurement, and LoRa wireless transmission. The graphene-based sensor exhibits stable low-frequency vibration responses and good linearity and can capture composite vibration signals containing 4 Hz and 50 Hz components. These results indicate the potential of the proposed system for future transmission-line vibration sensing applications.

## 1. Introduction

High-voltage transmission lines are influenced by different types of vibration induced by breeze, wind, and icing. The vibration generates alternating stress on conductors, insulators, fixtures, and even tower structures, inducing metal fatigue, wire strand breakage, bolt loosening, and insulator failure. Online vibration monitoring can be used to measure the vibration characteristics of the transmission lines, thereby detecting abnormal vibration in advance and avoiding permanent structural damage [1,2]. Although many different online vibration monitoring techniques have been proposed, the continuous power supply for distributed vibration sensor nodes remains one of the key bottlenecks that restrict their future development [3,4]. In addition, deployment safety under high-voltage conditions, low-power operation, and maintenance-free performance are also essential for these systems [5,6].

As for vibration sensing itself, the key requirement for transmission-line monitoring is sufficient low-frequency sensitivity. On the one hand, conductor galloping is usually characterized by low-frequency and large-magnitude wind-induced vibration, which is often associated with ice and wet-snow accretion that produces an asymmetric aerodynamic profile of the conductor. On the other hand, aeolian vibration is primarily induced by vortex shedding and is generally characterized by small-amplitude and relatively higher-frequency vibration. Consequently, practical transmission-line vibration is usually not governed by a single-frequency component, but rather appears as a composite dynamic signal consisting of low-frequency dominant motion and higher-frequency components [7,8,9,10]. For such signals, a vibration sensor should not only provide an effective response to low-frequency components but also preserve the high-frequency information contained in the composite vibration. Compared with conventional vibration-sensing methods, piezoresistive sensors generally offer a relatively simple structure, high sensitivity, and the capability to respond to low-frequency vibrations and even quasi-static loads [11,12,13]. As a representative two-dimensional material, graphene has been widely used in flexible piezoresistive mechanical sensors due to its excellent electrical conductivity, mechanical flexibility, and strain-dependent resistance variation [12]. On this basis, three-dimensional graphene aerogel forms a compressible conductive network, allowing external pressure, strain, or vibration to induce changes in inter-sheet contact area, conductive pathways, and electrical resistance. Previous studies have shown that graphene aerogel sensors are capable of detecting mechanical signals from low-frequency strain/pressure to higher-frequency vibration, which provides a material basis for transmission-line composite vibration monitoring [14]. The graphene-based vibration sensor employed in this study operates based on the piezoresistive effect, in which the resistance variation originates from changes in the internal conductive network induced by mechanical deformation. This sensing mechanism makes the sensor less dependent on charge accumulation or capacitive coupling and therefore provides a potential advantage for vibration sensing in electromagnetic field environments.

Based on this background, this study proposes a self-powered sensing system for transmission-line vibration monitoring, based on low-amplitude spatial magnetic field energy harvesting, duty-cycled self-powered operation, and low-frequency vibration sensing. From the energy-harvesting perspective, a spatial magnetic field energy harvester is designed to extract energy from the AC magnetic field around the transmission line, including low-amplitude magnetic field conditions. From the operational perspective, a duty-cycled circuit module is adopted to regulate the harvested energy and enable closed-loop self-powered operation of the system. From the sensing perspective, a graphene-based vibration sensor is employed to capture low-frequency and composite vibration signals. This study verifies the feasibility of the proposed concept from the aspects of energy harvesting, duty-cycled circuit operation, closed-loop self-powered operation, and vibration sensing. This study provides a basis for the future development of self-powered vibration sensing systems for transmission-line monitoring, while further long-term field validation and engineering optimization are still needed for practical deployment.

## 2. System Design

### 2.1. Concept of the System

**Figure 1:**
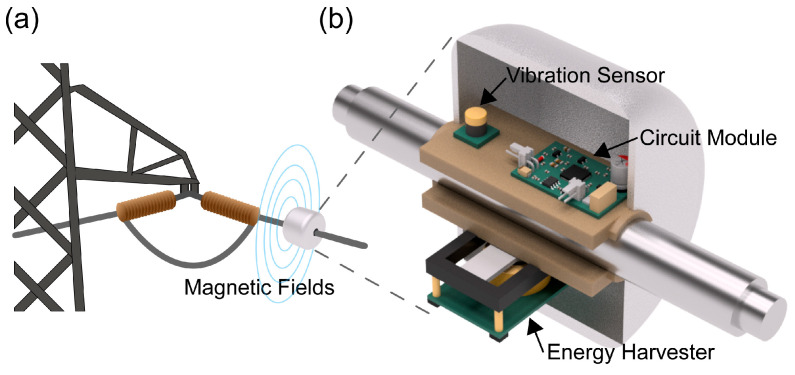
Schematic of the self-powered vibration sensing system. (**a**) Spatial magnetic field energy harvesting. (**b**) Internal configuration of the system.

The self-powered vibration sensing system is schematically illustrated in Figure 1. Figure 1a shows the application scenario in which the system harvests energy from the spatial magnetic field surrounding the transmission line. As the system is designed for vibration measurement of both aeolian vibrations and conductor galloping of the transmission line, a rigid and firm connection between the system and the transmission line must be established. Therefore, the metallic casing of the system is based on a tube structure that enables a direct clamp onto the high-voltage transmission lines. An electrical connection between all the components in the system and the transmission line is established through the metallic casing, in order to equalize their electric potential (e.g., 110 kV). This equipotential connection is essential for system operation as it effectively avoids phase-to-ground short-circuits caused by insulation failure. Figure 1b shows the internal configuration of the system. Three major modules of the monitoring system are mounted within the casing, namely, a spatial magnetic field energy harvester, a circuit module, and a graphene-based vibration sensor. The magnetic energy harvester collects power from the alternating spatial magnetic field (50 Hz) surrounding the transmission line. The graphene vibration sensor transforms the vibration signal to resistance change in the graphene. Compared with piezoelectric and capacitive sensors, the graphene aerogel vibration sensor is based on a piezoresistive mechanism rather than charge accumulation or capacitive coupling, which provides a potential advantage for vibration sensing in electromagnetic field environments. The resistance variation of the graphene originates from changes in the internal conductive network induced by mechanical deformation. This physical sensing mechanism endows the sensor itself with intrinsic immunity to strong magnetic noise. The circuit module mainly includes rectification, energy storage, voltage regulation, and switching control circuits. The power management circuit rectifies and regulates the output voltage from the energy harvester to provide a stable DC supply. Finally, the DC power drives the graphene vibration sensor to measure the vibration signal and transmit the data via LoRa.

### 2.2. Energy Harvester Design

**Figure 2:**
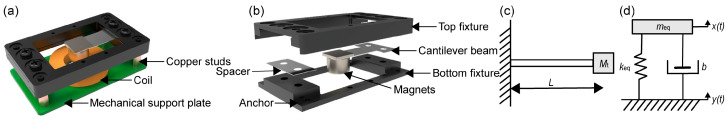
Structural design and simplified theoretical model of the magnetic field energy harvester. (**a**) Schematic view of the harvester. (**b**) Exploded view of the harvester structure. (**c**) Simplified cantilever beam with a tip mass. (**d**) Equivalent mass–spring–damper model.

As shown in Figure 2a, the magnetic field energy harvester consists of fixtures, a cantilever beam, magnets, a coil, and a mechanical support plate. The cantilever beam is fixed at one end, while the magnets are installed at the other end. The coil is placed beneath the magnets with a stable gap maintained by the support structure. The exploded view in Figure 2b further illustrates the assembly relationship between the fixtures, cantilever beam, magnets, coil, and support structure. The harvester has overall dimensions of 60 mm × 35 mm × 19.5 mm, and the cantilever beam measures 24 mm × 10 mm × 0.2 mm. The circular magnet has a diameter of 10 mm and a thickness of 7 mm, while the square magnet has a side length of 10 mm and a thickness of 1 mm. The coil has an inner diameter of 15 mm, an outer diameter of 29 mm, a height of 5 mm, a wire diameter of 0.16 mm, and 865 winding turns. The vertical distance between the cantilever beam and the coil is 9.5 mm. All of these geometric and coil parameters of the energy harvester are summarized in Table 1. To further clarify the theoretical basis of the harvester design, the cantilever–magnet structure is simplified as a cantilever beam with a tip mass, as shown in Figure 2c. The corresponding lumped mass–spring–damper model is shown in Figure 2d, where the cantilever–magnet structure is represented by the equivalent mass, equivalent stiffness, and damping coefficient. For this structure, the natural resonant frequency can be expressed as [15]:

$$f=\frac{1}{2\pi }\sqrt{\frac{k_{\mathrm{eq}}}{m_{\mathrm{eq}}}}=\frac{1}{2\pi }\sqrt{\frac{3EI}{\left(\frac{33}{140}M_{b}+M_{t}\right)L^{3}}}$$

where *k*_eq_ is the effective stiffness, *m*_eq_ is the effective mass, *EI* is the flexural rigidity of the cantilever beam, *L* is the beam length, *M*_b_ is the mass of the cantilever beam, and *M*_t_ is the tip mass. Based on the structural parameters, the calculated natural frequency is 51.87 Hz, which is close to the experimentally measured resonant frequency of 50.00 Hz. This result indicates that the cantilever–magnet structure is designed to operate at a frequency close to 50 Hz. The slight deviation between the calculated and measured resonant frequencies is mainly attributed to model simplifications, material property deviations, fabrication tolerances, and slight geometrical asymmetries introduced during assembly.

The magnetic field energy harvester operates based on magnetic field-induced vibration and electromagnetic induction. When the harvester is placed in the 50 Hz alternating magnetic field surrounding the transmission line, the field acts on the magnets and generates periodic magnetic forces, driving the cantilever beam and magnets to vibrate. The relative motion between the magnets and the coil changes the magnetic flux through the coil, leading to an induced voltage across the coil and enabling the conversion of magnetic field energy into electrical energy. This design achieves non-contact energy harvesting from the transmission line, making it suitable for equipotential installation. At the same time, the cantilever–magnet design is able to collect energy across a wide current range. However, its output characteristics are affected by the magnetic field strength, installation position, magnetic field direction, natural frequency of the cantilever structure and distance between the magnet and the coil; therefore, these factors should be considered when adapting the harvester to different transmission-line operating conditions and installation scenarios.

### 2.3. Vibration Sensor Design

**Figure 3:**
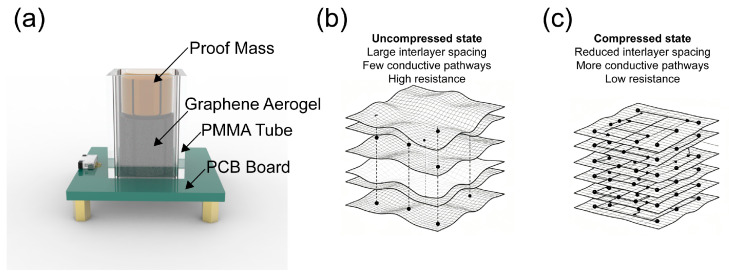
Structure and piezoresistive mechanism of the graphene aerogel vibration sensor. (**a**) Structural schematic. (**b**) Uncompressed state with few conductive pathways and high resistance. (**c**) Compressed state with more conductive pathways and low resistance.

As shown in Figure 3a, the graphene aerogel vibration sensor design employs a sandwich structure, consisting of a proof mass on the top, the graphene aerogel in the middle, and two electrodes on the bottom PCB board. A PMMA tube is employed to constrain the lateral motion of the proof mass and the graphene aerogel, thereby minimizing off-axis error. As illustrated in Figure 3b,c, the response of the graphene aerogel vibration sensor originates from the piezoresistive effect of its porous lamellar structure. In the uncompressed state, the large interlayer spacing results in fewer contact points and conductive pathways, leading to high resistance. Under external vibration, the inertial force of the proof mass periodically compresses the graphene aerogel, reducing the interlayer spacing, increasing the contact area, and forming more conductive pathways; as a result, the resistance decreases. In this way, external vibration can be converted into a periodic resistance variation. Unlike piezoelectric vibration sensors that depend on the dynamic generation of charges, the output of the graphene aerogel vibration sensor depends on deformation-induced resistance changes, ensuring effective responses under low-frequency vibration excitation and even quasi-static loading. Meanwhile, the low Young’s modulus and high compressibility of the graphene aerogel enable distinct deformation even under small-amplitude vibrations, leading to superior low-frequency vibration sensing capability. Although the graphene aerogel sensor has advantages such as excellent mechanical compliance, low-power consumption, and good low-frequency vibration response, several challenges should also be considered. In the proof mass/graphene aerogel/electrode sandwich structure, lateral displacement or tilting of the proof mass may introduce off-axis error during vibration sensing. In addition, the intrinsic softness and porous microstructure of the graphene aerogel may cause viscoelastic hysteresis, baseline drift, and mechanical relaxation under repeated compression. These factors highlight the importance of structural constraint and signal calibration for achieving stable and repeatable sensor responses.

### 2.4. Circuit Design and System Integration

**Figure 4:**
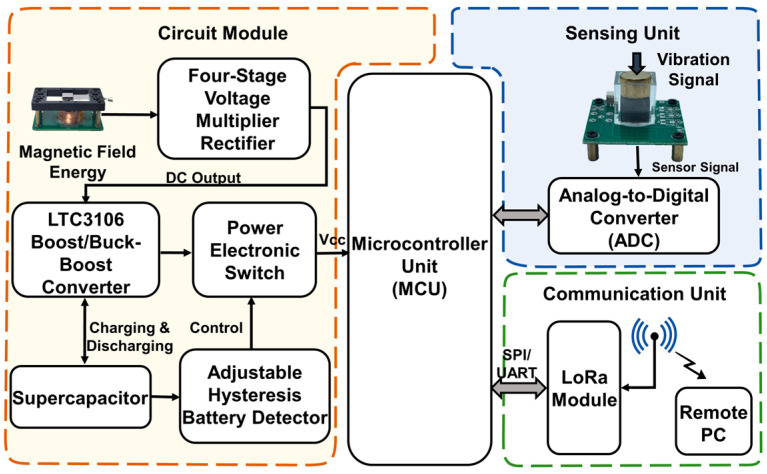
Hardware topology of the duty-cycled circuit module and system integration.

Figure 4 shows the hardware topology of the circuit module and the system loads. The system loads include the graphene-based vibration sensor, sensing circuit, STM32L496 microcontroller, and ATK-LoRa wireless transmission module. The harvested alternating power is first processed by a four-stage voltage-multiplier rectifier to boost the voltage and convert it into DC power, after which the LTC3106 converter stores the harvested energy in a supercapacitor. To ensure stable operation, an adjustable hysteretic battery detector is introduced to control the power switch. This system not only enables energy accumulation over time, but also isolates the ultra-low-power energy-harvesting side from the highly dynamic load side, thereby ensuring the stability of the operating voltage even under transient current demands from the sensing and communication units.

## 3. Experimental Procedures

### 3.1. Materials

Graphene oxide (GO) aqueous dispersion (5 mg/mL, product code XF224-1, with a flake size of 0.5–5 µm, a thickness of 1–6 nm, a purity of >99%, and 1–6 layers) is purchased from Nanjing XFNANO Materials Tech Co., Ltd. (Nanjing, China). L-Ascorbic acid (product code A103534) is purchased from Aladdin Industrial Corporation (Shanghai, China). All other chemical reagents are of analytical grade and are used as received without further purification.

### 3.2. Preparation of Graphene Aerogel

The graphene aerogel is fabricated based on a previously reported method [16], with some modifications. In total, 15 mg of L-ascorbic acid is added to 1.5 mL of the GO aqueous dispersion (5 mg/mL). The mixture is vortexed and then heated in a boiling water bath for 8–12 min. Afterwards, the sample is directionally frozen in liquid nitrogen for 30 min. Then, the sample is freeze-dried using a freeze-dryer (Christ Alpha 1-2 LD plus, Martin Christ Gefriertrocknungsanlagen GmbH, Osterode am Harz, Germany) at −47 °C and a pressure of 0.2 mbar. Finally, the obtained aerogel is thermally annealed in a tube furnace (OTF-1200X, Hefei Kejing Material Technology Co., Ltd., Hefei, China). The sample is heated to 1000 °C at a rate of 5 °C/min under argon flow, maintained for 1 h, and then allowed to cool naturally to room temperature.

### 3.3. Fabrication of the Sensor

First, the graphene aerogel is positioned on the bottom electrodes of the PCB board to establish a conductive pathway between the two electrodes. After the sensing element is positioned, the PMMA tube is aligned with the electrode region and bonded to the PCB board, providing lateral confinement for the graphene aerogel and the proof mass. Finally, the proof mass is mounted on top of the graphene aerogel to enhance the inertial compression applied to the sensing element under external vibration. This fabrication process forms a proof mass/graphene aerogel/electrode sandwich structure, and the electrical signal is read out through the PCB pads.

### 3.4. Characterization and Measurement Setup

**Figure 5:**
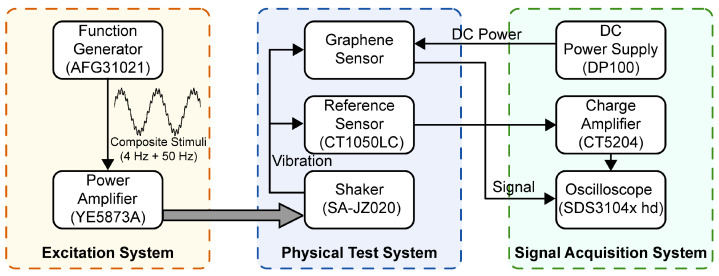
Characterization and measurement setup for the graphene aerogel vibration sensor.

The characterization and measurement setup of the graphene aerogel vibration sensor is shown in Figure 5. It consists of an excitation system, a physical test system, and a signal acquisition system. First, a function generator (AFG31021, Tektronix, Inc., Beaverton, OR, USA) generates the vibration excitation signal, which is amplified by a power amplifier (YE5873A, Sinocera Piezotronics Inc., Yangzhou, China) and then sent to a shaker (SA-JZ020, Wuxi Shi’ao Technology Co., Ltd., Wuxi, China). In this study, a 4 Hz vibration signal is employed to represent low-frequency conductor-galloping-like motion, while a composite signal consisting of 4 Hz and 50 Hz components is used to evaluate the sensor response under superposed low-frequency and higher-frequency vibration components. Subsequently, the graphene sensor and a reference sensor (CT1050LC, Shanghai Chengtec Electronics Co., Ltd., Shanghai, China; sensitivity: 500 mV/g, measurement range: ±10 g, frequency range: 0.2 Hz–3 kHz, resonant frequency: 5 kHz, resolution: 0.1 mg, nonlinearity: ±0.1%) are installed coaxially on the shaker to ensure identical mechanical vibration inputs. The signal acquisition system consists of a DC power supply (DP100, Guangzhou Xingyi Electronic Technology Co., Ltd., Guangzhou, China), a charge amplifier (CT5204, Shanghai Chengtec Electronics Co., Ltd., Shanghai, China), and an oscilloscope (SDS3104X HD, SIGLENT Technologies Co., Ltd., Shenzhen, China). The DC power supply provides the operating voltage for the graphene aerogel sensor. For the graphene aerogel sensor, the resistance variation is converted into a voltage variation through a voltage-divider circuit and then recorded as the voltage output. During calibration, this output voltage is synchronously compared with the acceleration measured by the CT1050LC reference sensor, and the obtained sensitivity is used to convert the sensor output into acceleration. The charge amplifier is used to condition and amplify the output signal from the reference sensor. The output signals from both the graphene sensor and the reference sensor are synchronously recorded by the oscilloscope.

The uncertainty of the vibration sensing measurement is evaluated based on the reference acceleration and the output voltage of the graphene aerogel sensor. The reference acceleration is calculated as a_ref_ = V_ref_/(K_ref_ × G), where K_ref_ = 0.5 V/g represents the sensitivity of the CT1050LC reference sensor, and G = 1 represents the gain of the CT5204 signal conditioner. The sensitivity of the graphene aerogel sensor is calculated as S = V_GA_/a_ref_. The measurement uncertainty mainly originates from the reference sensor sensitivity error of ±2%, the CT5204 signal conditioner accuracy error of <1%, the SDS3104X HD oscilloscope vertical measurement accuracy of ±1%, the nonlinear error of the reference sensor of ±0.1%, and the waveform repeatability error obtained from cycle-by-cycle amplitude variations over a 10 s acquisition window. For the 50 Hz vibration test, this acquisition window corresponds to 500 vibration cycles. By combining these independent uncertainty components using the root-sum-square method, the relative combined uncertainty of the sensing measurement is estimated to be 3.359%.

## 4. Results

### 4.1. Fabrication Results

**Figure 6:**
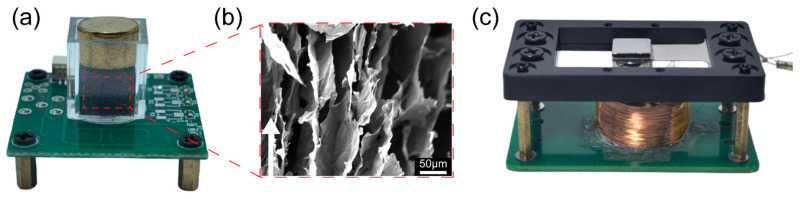
Fabrication results of the graphene aerogel vibration sensor and magnetic field energy harvester. (**a**) Fabricated graphene aerogel vibration sensor. (**b**) SEM image of the graphene aerogel. (**c**) Fabricated magnetic field energy harvester.

Figure 6 shows the fabrication results of the graphene aerogel vibration sensor and magnetic field energy harvester. Figure 6a shows the assembled graphene aerogel vibration sensor, where the proof mass, graphene aerogel, PMMA tube, and PCB board are well aligned and integrated. For the assembled sensor, the copper proof mass is 3.52 g, corresponding to a gravitational preload of approximately 34.5 mN. The graphene aerogel has an original diameter of 8.9 mm and height of 9.32 mm and is pre-compressed to a working height of 7.12 mm after assembly, giving an initial sensing resistance of approximately 112.7 Ω. As shown in Figure 6b, the graphene aerogel exhibits an internally aligned lamellar and porous structure. This highly porous micro-skeleton provides excellent mechanical compliance and sufficient compressible space, which forms the structural basis for its piezoresistive behavior. Figure 6c shows the assembled magnetic field energy harvester, in which the cantilever beam, magnets, coil, and support structure are compactly integrated.

### 4.2. Dynamic Behavior of the Energy Harvester

**Figure 7:**
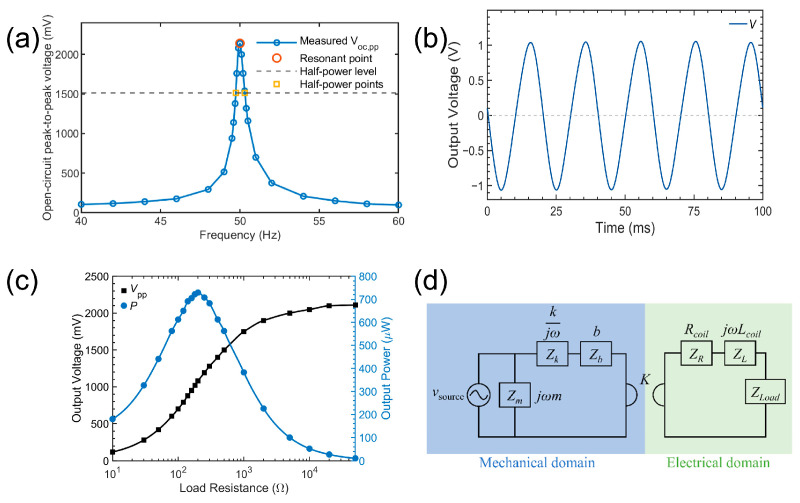
Experimental results and equivalent-circuit model of the energy harvester. (**a**) Frequency response of the open-circuit peak-to-peak output voltage. (**b**) AC output voltage waveform under 0.11 mT magnetic field excitation at 50 Hz. (**c**) Output voltage and output power as a function of load resistance. (**d**) Equivalent-circuit model of the energy harvester for impedance-matching analysis.

The output characteristics of the magnetic field energy harvester under 0.11 mT magnetic field excitation at 50 Hz are shown in Figure 7. Based on the 5 cm distance between the transmission-line center and energy harvester center in the current installation setup, together with a light-load current of 28 A, a characteristic magnetic flux of 0.11 mT can be estimated and is selected as a representative of light-load magnetic field strength. In transmission-line energy-harvesting applications, conventional current-transformer (CT)-based energy harvesters cannot operate effectively under low-current conditions. To identify the resonance characteristics of the harvester, the open-circuit peak-to-peak output voltage is measured as a function of excitation frequency, as shown in Figure 7a. The output voltage reaches a maximum value of 2140 mV at 50.00 Hz. Based on the half-power criterion, the corresponding half-power voltage level is 1513.21 mV, and the half-power frequencies are 49.735 Hz and 50.312 Hz. Therefore, the measured bandwidth is 0.577 Hz, giving a quality factor of 86.64 and a damping ratio of 0.00577. Based on the identified equivalent mass and stiffness, the equivalent viscous damping coefficient is calculated to be 0.01715 N·s/m. These results further confirm that the harvester is designed to operate near the 50 Hz power–frequency magnetic field. In contrast, as shown in Figure 7b, the proposed energy harvester generates a stable sinusoidal AC voltage output under a low-amplitude magnetic field of 0.11 mT at 50 Hz. To evaluate the practical load capacity and determine the optimal operating range of the energy harvester, a load-matching experiment is conducted. Figure 7c presents the variations in peak-to-peak output voltage and output power with external load resistance. As the load resistance increases over a wide range, the output voltage increases monotonically and gradually approaches the open-circuit saturation value, while the output power exhibits a distinct peak. Although the coil has a DC resistance of 50 Ω, the results suggest that the effective dynamic internal impedance is significantly higher under 50 Hz AC operation. The measured curves show that the maximum output power is achieved when the external resistance is 200 Ω, indicating that impedance matching is attained at this point. To explain this impedance-matching behavior, the equivalent-circuit model of the energy harvester is shown in Figure 7d, where the mechanical and electrical domains are coupled through the electromechanical coupling coefficient *K*. According to the equivalent-circuit representation of the magnetic field energy harvester, maximum power transfer is achieved when the load impedance is matched to the complex conjugate of the equivalent internal impedance of the harvester [17]:

$$Z=R_{\mathrm{coil}}+\frac{K^{2}b\omega^{2}}{{\left(k_{\mathrm{eq}}-m_{\mathrm{eq}}\omega^{2}\right)}^{2}+b^{2}\omega^{2}}-j\left[\frac{K^{2}\omega \left(k_{\mathrm{eq}}-m_{\mathrm{eq}}\omega^{2}\right)}{{\left(k_{\mathrm{eq}}-m_{\mathrm{eq}}\omega^{2}\right)}^{2}+b^{2}\omega^{2}}+L_{\mathrm{coil}}\omega \right]$$

where *K* is the electromechanical coupling coefficient; *m*_eq_, *b*, and *k*_eq_ are the effective mass, viscous damping coefficient, and effective stiffness in the mechanical domain, respectively; and *R*_coil_ and *L*_coil_ are the coil resistance and coil inductance, respectively. This model indicates that the equivalent internal resistance under resonant operation is not determined only by the coil resistance, but also includes the reflected electromechanical resistance introduced by mechanical damping and electromechanical coupling. Therefore, the additional reflected electromechanical resistance is estimated as *R*_opt_ − *R*_coil_ ≈ 150 Ω. Using the identified damping coefficient, the effective electromechanical coupling coefficient is estimated to be approximately 1.60 N/A, or equivalently 1.60 V·s/m. This explains why the optimal load resistance is approximately four times larger than the coil DC resistance. Similar differences between the optimal load resistance and the coil resistance have also been reported in previous studies on energy harvesters [18]. Under this condition, the harvester delivers a maximum output power of around 729 μW. This ability to harvest more than 700 μW under a conservative 0.11 mT light-load magnetic field condition demonstrates its potential for weak-field energy harvesting and provides a sufficient power margin for subsequent circuit modules.

### 4.3. Power Management and Energy Autonomous Operation

**Figure 8:**
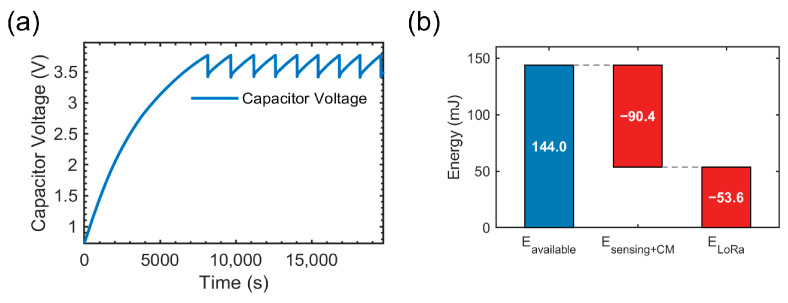
Energy-autonomous operation of the proposed system. (**a**) Capacitor voltage profile during harvester-powered sensing and wireless transmission. (**b**) Energy breakdown for a single operation cycle. CM denotes the circuit module.

Although the proposed energy harvester achieves a maximum output power of 729 μW, this low continuous power is insufficient to directly drive the microcontroller unit, wireless communication module, and sensing unit. Therefore, the duty-cycled circuit module described above is further evaluated to verify the autonomous operation of the vibration sensing system. The terminal voltage of the supercapacitor during the operation is shown in Figure 8a. In this experiment, no external DC power supply is used, and the harvested energy from the magnetic field energy harvester is used to power all system loads, including the graphene-based vibration sensor, sensing circuit, MCU, and LoRa wireless transmission module. Starting from a low initial voltage of around 0.8 V, the 100 mF supercapacitor undergoes a cold-start charging process before entering the stable duty-cycled operation stage. Subsequently, the circuit module operates in a typical duty-cycled mode with a sawtooth-like voltage profile. The supercapacitor voltage rises gradually during the steady-stage energy accumulation stage. Once it reaches the predefined wake-up voltage threshold of 3.8 V, the circuit module executes the scheduled sensing and communication tasks and returns to the recharging phase. The energy stored in or consumed from the supercapacitor is calculated from its voltage variation according to

$$E=\frac{1}{2}C\left(V_{\mathrm{start}}^{2}-V_{\mathrm{end}}^{2}\right)$$

where C is the capacitance of the supercapacitor, and $V_{\mathrm{start}}$ and $V_{\mathrm{end}}$ are the capacitor voltages before and after the corresponding operation stage, respectively.

To evaluate the energy-autonomous operation capability of the system, Figure 8b presents the updated energy breakdown for a single operation cycle. During one duty cycle, the available energy obtained from the 100 mF supercapacitor discharge from 3.8 V to 3.4 V is 144.0 mJ. The energy consumed by sensing and circuit-module operation is estimated to be 90.4 mJ, while the additional energy associated with LoRa wireless transmission is 53.6 mJ. By comparing the discharge curves with and without LoRa transmission, the additional power associated with LoRa-enabled wireless transmission is estimated to be approximately 117 mW. This result demonstrates that the harvested energy stored in the supercapacitor is sufficient to support vibration sensing, circuit-module operation, MCU operation, and LoRa wireless transmission within one closed-loop operation cycle. In addition, LoRa communication is adopted in this system because it provides a favorable balance between low energy consumption and long-range wireless transmission. In typical open environments of overhead transmission-line corridors, LoRa communication can provide a transmission range of several kilometers, which is suitable for distributed monitoring in power-grid applications. Therefore, the measured LoRa energy consumption of 53.6 mJ per duty cycle indicates that the proposed duty-cycled operation can support not only vibration sensing and circuit-module operation but also low-power long-range wireless communication.

### 4.4. Characteristics of the Graphene Aerogel Vibration Sensor

**Figure 9:**
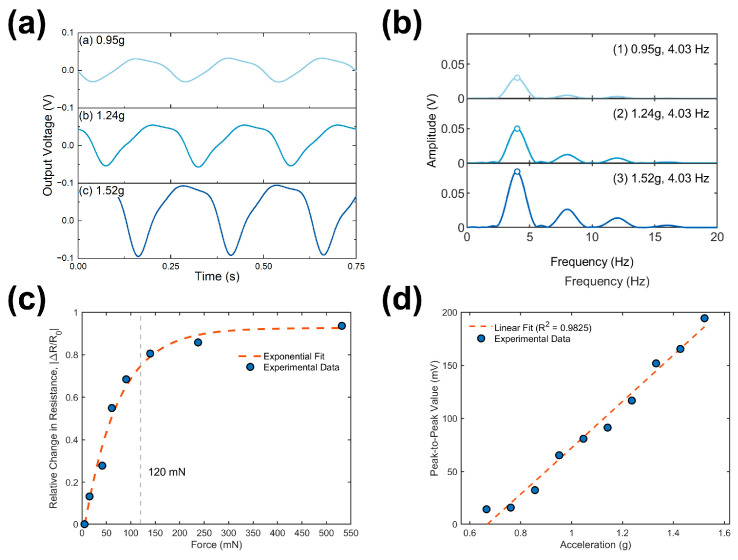
Sensing characteristics of the graphene aerogel vibration sensor. (**a**) Dynamic output voltage waveforms and (**b**) their corresponding frequency-domain spectra under various accelerations. (**c**) Relative change in resistance as a function of applied force. (**d**) Linear fit of the peak-to-peak output voltage versus acceleration.

Figure 9 shows fundamental sensing characteristics of the graphene aerogel vibration sensor. The dynamic sensing characteristics of the sensor are evaluated by measuring its output voltage waveforms under different acceleration levels (0.95 g, 1.24 g, and 1.52 g) at 4 Hz, as shown in Figure 9a. The waveforms exhibit stable periodic behavior, and the signal amplitudes increase with increasing acceleration. These results confirm that the sensor can maintain a stable electrical response under low-frequency vibration excitation. To further validate the accuracy of the frequency response of the sensor, FFT analysis is applied to the waveforms shown in Figure 9a, and the results are presented in Figure 9b. The spectra clearly show that the dominant frequency remains accurate and stable at 4.03 Hz under different acceleration levels, corresponding well to the applied excitation frequency. This result confirms the capability of the sensor to accurately capture low-frequency vibration signals. Furthermore, the static force response of the sensor is characterized by measuring the relative resistance change ($\left|\Delta R/R_{0}\right|$) under different applied forces, as shown in Figure 9c. As the applied force increases from 0 to 120 mN, the relative resistance change rises rapidly. Beyond 120 mN, the relative resistance change gradually approaches saturation and reaches a maximum value of around 0.9. This result indicates that the graphene aerogel is a promising piezoresistive material for vibration sensing. The peak-to-peak values of the output voltage waveforms are extracted and correlated with the excitation acceleration, as shown in Figure 9d. Over the acceleration range from 0.7 g to 1.5 g, the sensor exhibits excellent linearity, with a coefficient of determination $R^{2}=0.9825$. These results indicate that graphene aerogel is a promising material for low-frequency vibration sensing.

### 4.5. Sensing Performance Under Simulated Field-Oriented Composite Vibration Stimuli

**Figure 10:**
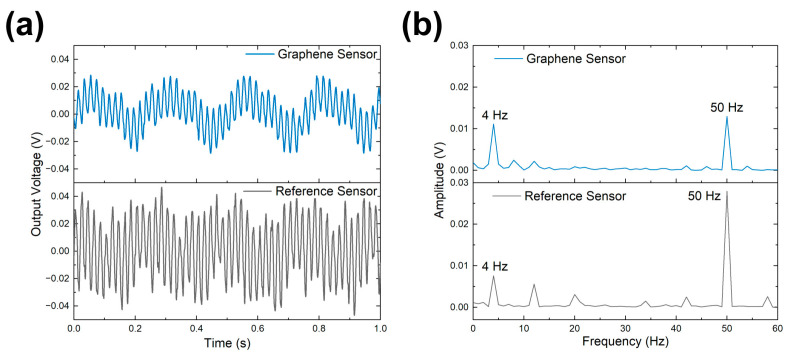
High-fidelity sensing performance and analysis under composite vibration stimuli: (**a**) time-domain output voltage waveforms and (**b**) corresponding frequency-domain spectra of the graphene sensor and the commercial reference sensor under a 4 Hz + 50 Hz composite excitation.

In practical smart-grid environments, the vibration of transmission lines is usually not a single ideal sinusoidal waveform but rather a composite signal formed by the superposition of vibrations at different frequencies. To evaluate the sensing performance of the proposed system under a simulated field-oriented composite vibration condition, a composite excitation signal containing 4 Hz and 50 Hz components is applied to the shaker through the excitation system to simulate complex vibrations in practical applications. The 4 Hz component is used to represent a low-frequency conductor-motion or swing component, whereas the 50 Hz component is introduced as a representative higher-frequency vibration component superimposed on the low-frequency motion. The output voltage waveforms of the graphene sensor and the reference sensor are shown in Figure 10a. As can be seen, the waveform of the graphene sensor retains the low-frequency envelope at 4 Hz while smoothly superimposing the 50 Hz high-frequency component, thereby reflecting the composite physical displacement of the shaker. In contrast, the waveform of the reference piezoelectric sensor shows no clear low-frequency pattern and is dominated by high-frequency oscillations, resulting in a severely blurred baseline. To further validate the time-domain observations, FFT analysis is performed on the output signals, as shown in Figure 10b. In the frequency-domain spectrum, the 4 Hz and 50 Hz characteristic peaks are clearly resolved by the graphene aerogel sensor, indicating that vibration components at different frequencies are effectively captured. In contrast, the frequency-domain spectrum of the reference piezoelectric sensor is dominated by the 50 Hz response, whereas the signal at 4 Hz is strongly attenuated. These frequency-domain results are highly consistent with the time-domain waveforms, and the high-fidelity low-frequency vibration sensing capability of the graphene aerogel sensor is demonstrated in comparison with the reference piezoelectric sensor. Conventional piezoelectric sensors are inherently limited at low frequencies because of charge leakage, and the fact that the acceleration amplitude of a given sinusoidal displacement decreases rapidly at lower frequencies ($a\propto f^{2}$). In contrast, the graphene sensor maintains an effective response in the low-frequency range. Owing to its piezoresistive sensing mechanism, the graphene sensor retains sensitivity to low-frequency vibrations and even static loading. These results support the potential of the graphene-based sensor for tracking multi-component vibration signals under simulated field-oriented laboratory excitation.

### 4.6. Stability and Durability Analysis

**Figure 11:**
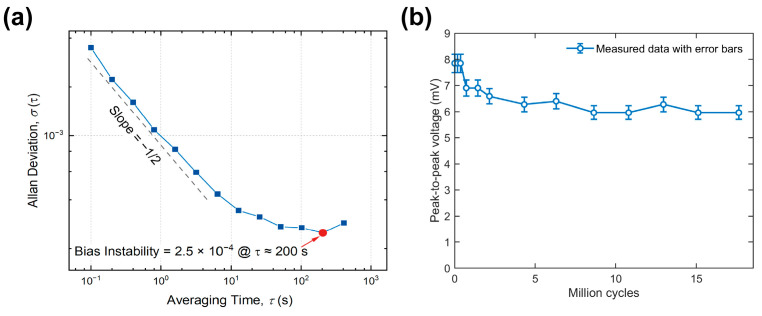
Stability and durability characterization of the graphene aerogel vibration sensor: (**a**) Allan deviation analysis; (**b**) cyclic durability test at 50 Hz and 0.5 g.

Figure 11a shows the double-logarithmic plot of acceleration-equivalent Allan deviation $\sigma \left(\tau \right)$ as a function of averaging time τ. The voltage-domain Allan deviation is converted into acceleration-equivalent values using the 4 Hz sensitivity of 218.6 mV/g obtained from the linear calibration results. At short averaging times, the curve exhibits a typical slope of −1/2. This behavior indicates that the sensor noise is dominated by white noise in this time range, which can be effectively suppressed by temporal averaging or low-pass filtering. With increasing averaging time, the curve gradually decreases and reaches its minimum value. The results indicate that at an averaging time of around 200 s the graphene aerogel sensor reaches a minimum acceleration-equivalent bias instability of $2.5\times 10^{-4}\;g$. This suggests low baseline drift within the current test window.

To further evaluate the cyclic stability of the graphene aerogel sensor, a durability test is conducted under 50 Hz and 0.5 g cyclic vibration, as shown in Figure 11b. The sensor is continuously tested for 17.64 million cycles. The peak-to-peak voltage decreases during the initial stage, especially within the first 8.64 million cycles, and then reaches a relatively stable level. The error bars indicate the measurement error range of the extracted peak-to-peak voltage, and the calculation method is consistent with the uncertainty analysis described in Section 3.4. The results show that the graphene aerogel sensor undergoes an initial settling process under repeated compression, followed by a relatively stable working state. These characteristics mainly originate from two aspects: firstly, cyclic vibration induces slight irreversible deformation and interlayer sliding of graphene sheets within the aerogel structure, which subsequently increases the internal resistance; secondly, the residual stress inside the material is continuously released during early cycling. Although the device shows relatively robust behavior against cyclic fatigue after stabilization, the initial drift may have detrimental effects on the sensor’s accuracy during practical deployment. Therefore, the sensor should be carefully calibrated to remove the measurement artifact induced by the drift. However, time-dependent calibration over a long drifting period is challenging because of the need for time- or cycle-counting circuitry/algorithms and the behavioral discrepancies between different sensor devices. A burn-in cycling process may be performed before field installation to settle the structure of the graphene aerogel, but a lengthy burn-in process for each sensor is also impractical. Therefore, the key to improving the initial drift of the sensor is to suppress the structural variation of the graphene aerogel under cyclic excitation. This can be implemented in future work by optimizing the fabrication process and process parameters to increase the cross-linking of the aerogel structure, performing short burn-in and annealing processes to remove the majority of the initial drift, implementing a time/operation-history-based calibration algorithm with the assistance of the counter in the MCU, and developing dedicated packaging technologies to protect the aerogel, electrodes, and contacts from further early-stage degradation.

### 4.7. Validation of the Closed-Loop Self-Powered Operation

To validate the self-powered capability of the proposed system, a closed-loop self-powered operation experiment is conducted. During the test, no external DC power supply is used. All of the required energy is harvested from the environmental magnetic field, rectified by a four-stage voltage multiplier rectifier, and stored in a 100 mF supercapacitor. The stored energy is then regulated by the LTC3106 buck-boost DC/DC converter to provide a stable output voltage with high efficiency. Meanwhile, an adjustable hysteretic battery detector is introduced to control the on/off state of the power switch, thereby enabling duty-cycled dynamic energy management. The regulated voltage from the circuit module is used as the sole power source for the graphene-based vibration sensor, sensing circuit, STM32L496 microcontroller, and ATK-LoRa wireless transmission module, thereby directly verifying the cooperative operation of the spatial magnetic field energy harvester, the circuit module, and the sensing front end.

**Figure 12:**
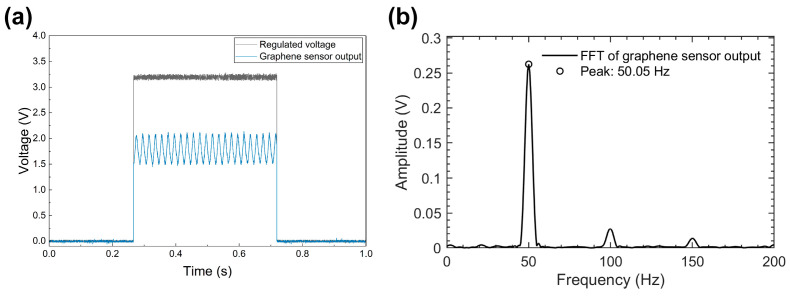
Performance of the closed-loop self-powered operation: (**a**) dynamic waveforms of the regulated supply voltage (black) and the graphene-based sensor output signal (blue); (**b**) Fast Fourier Transform (FFT) spectrum of the sensor output, indicating a peak frequency at 50.05 Hz.

During the experiment, a 50 Hz vibration excitation is applied by the shaker to simulate the operating vibration condition of a transmission line. The waveforms of the regulated voltage (black line) and the graphene sensor output (blue line) under closed-loop self-powered operation are shown in Figure 12a. When the supercapacitor terminal voltage reaches the predefined turn-on threshold, the regulator output is enabled and rapidly rises to approximately 3.2 V at 0.26 s. During the subsequent approximately 0.45 s powered operation window, the stored energy in the supercapacitor is released to support the sensing, circuit-module operation, MCU operation, and wireless transmission process. Within this powered operation window, the graphene-based sensor output presents a continuous 50 Hz waveform that is consistent with the applied excitation, confirming successful vibration sensing under fully self-powered conditions. Moreover, the Fast Fourier Transform (FFT) spectrum of the sensor output, as shown in Figure 12b, exhibits a distinct peak at 50.05 Hz, which precisely corroborates the 50 Hz applied excitation frequency. Notably, the approximately 0.45 s powered operation window demonstrates the reliable cooperative operation of the energy harvester, circuit module, sensing unit, MCU, and wireless transmission module. Combined with the energy budget analysis in Section 4.3, this result confirms that the harvested energy stored in the supercapacitor is sufficient to support vibration sensing, circuit-module operation, MCU operation, and LoRa wireless transmission within one closed-loop operation cycle.

This quantitatively proves that the stored energy is able to sustain the complete process of vibration sensing and wireless data transmission. In addition, from the perspective of hardware scalability, if more complex algorithms or other advanced functions are required, the capacitance of the supercapacitor can be increased to store more energy, although this will correspondingly increase the charge time of the supercapacitor. These results demonstrate that the proposed sensing front end is able to work without an external power source, thereby verifying its closed-loop self-powered capability for vibration measurement using harvested environmental energy.

## 5. Discussion

This study proposes a duty-cycled self-powered vibration sensing system for transmission-line monitoring, which integrates energy harvesting from a low-amplitude magnetic field, and closed-loop self-powered operation. The equipotential system enables the device to be directly clamped onto the transmission line and maintained at the same electric potential as the conductor, thereby reducing phase-to-ground short-circuit risks caused by insulation failure. Meanwhile, the spatial magnetic field energy harvester can generate AC output under 0.11 mT magnetic field excitation at 50 Hz. Further impedance-matching results show that the energy harvester achieves a maximum output power of 729 μW at an external resistance of 200 Ω. With the duty-cycled circuit module, the energy harvester is able to drive the MCU, the communication unit, and the sensing unit through a duty-cycled operation. For the sensing perspective, the graphene aerogel vibration sensor exhibits good low-frequency vibration sensing performance. The results show that, under 4 Hz vibration, the graphene sensor produces stable output waveforms and good linearity over the acceleration range from 0.7 g to 1.5 g. Under composite excitation consisting of 4 Hz and 50 Hz sinusoidal components, the graphene sensor preserves both the 4 Hz envelope and 50 Hz sinusoidal components, indicating its capability to capture composite vibration signals under the tested conditions.

Table 2 further compares this work with state-of-the-art systems in terms of energy source, harvester mechanism, power characteristics, operating condition, sensing element, and deployment system. Unlike kinetic-energy harvesters that are highly dependent on specific wind speeds or galloping conditions [4,19,20,21], the proposed system harvests energy from the line-current-induced spatial AC magnetic field and achieves a maximum output power of 729 μW under a weak magnetic flux density of 0.11 mT. Compared with the magnetic field energy harvester in [22], the proposed harvester provides a higher output power under weak magnetic field excitation. Compared with the CT-based system [23], the proposed mechanical harvester harvests the spatial magnetic field; therefore, no complete magnetic field loop needs to be formed. Although the CT-based system in [23] can operate from 1 A to 100 A, CT harvesters are still highly dependent on the line current and magnetic-core design. A CT harvester designed for low-current operation may suffer from magnetic-core saturation, excessive heating, and large voltage fluctuation under high-load or surge-current conditions. In contrast, the proposed harvester is designed to oscillate under a weak magnetic field, and the resonating amplitude of the magnet can be limited by a soft mechanical stopper structure. From the perspective of the deployment system, unlike the commonly used insulated suspension configuration in existing self-sensing systems [4,19,22], the equipotential clamping design used in this work reduces the requirement for additional insulation design and mitigates insulation-related short-circuit risks.

Although this study demonstrates the feasibility of the proposed system in weak AC magnetic field energy harvesting, low-frequency and composite vibration sensing, and closed-loop self-powered operation, several issues still require further investigation for long-term practical applications. First, the present experiments are mainly conducted under laboratory conditions, including controlled magnetic field excitation, shaker-based vibration input, and short-term closed-loop self-powered testing. In real transmission-line environments, the magnetic field strength, vibration mode, ambient conditions, and installation state may vary with operating conditions. In addition, although the possible temperature and humidity effects have been discussed based on previous studies, direct environmental characterization of the integrated vibration sensing system has not yet been performed in this work. Temperature variation may affect the sensitivity of the graphene aerogel sensor [24], the resonance and output characteristics of the magnetic field energy harvester, and the leakage behavior of the supercapacitor, while humidity may also influence packaging reliability and long-term electrical stability [25]. Therefore, future field-deployment studies should include systematic temperature–humidity testing of the integrated system, together with temperature compensation, environmental calibration, and thermal/moisture-resistant encapsulation strategies. Therefore, long-term field evaluation under representative transmission-line operating conditions is still required for future engineering deployment. Second, although the proposed spatial magnetic field energy harvester avoids several CT-related limitations, the output power of the current prototype is still lower than that of conventional CT-based harvesters. Therefore, the proposed system is designed for duty-cycled operation. As a proof-of-concept demonstrator, the proposed energy-harvesting system is designed to work under conservative conditions of low current and weak magnetic field. In this work, the energy harvester is evaluated under a 0.11 mT magnetic field excitation, which corresponds to the magnetic field at 5 cm from a conductor carrying a current of approximately 28 A. Therefore, the tested condition represents a conservative case for evaluating the proposed duty-cycled self-powered operation. For practical transmission-line monitoring, aeolian vibration and galloping failures are often cumulative processes with continuous development, which often evolve over a relatively long time; therefore, very-high-frequency measurement is not needed for most cases. To monitor the line vibration with low power consumption, aeolian vibration can be measured at a 30 min interval under normal conditions and at a 10 min interval in high-risk sections; once excessive change in the vibration amplitude or frequency is detected, the measurement interval will be shortened to 1 min. For galloping monitoring, the regular measurement interval is around 10 min, which is changed to 1–2 min in icing seasons and icing-prone areas, and continuous monitoring will be enabled immediately after galloping is identified. In addition, data transmission is not necessary for every measurement in the normal monitoring operation. Instead, the amplitude and frequency data can be temporarily stored in the MCU and transmitted every 6 h or so. This further reduces the power needed for normal monitoring. Therefore, the proposed self-powered sensor, served as a proof-of-concept prototype, can meet the vibration monitoring requirements in a normal status. Surplus energy might even be realized by using short-interval measurement, temporary data storage, and long-interval wireless data transmission. However, the device is not capable of conducting high-frequency (e.g., 1 min interval measurement and transmission) or continuous monitoring. To address this issue, the proposed sensor system must be further optimized, i.e., by increasing the capacity of supercapacitors and increasing the volume of magnetic field energy harvesters for higher energy-harvesting capability. Third, as shown in Table 3, the proposed graphene aerogel sensor shows comparable or higher sensitivity compared with current research-grade graphene vibration sensors [14,22] and is evaluated over 17.64 million cycles. However, although the peak sensitivity of the graphene aerogel sensor (625 mV/g) is higher than that of the commercial piezoelectric accelerometer, the validated frequency range (4–500 Hz) and linear measurement range (0.7 g–1.5 g) are narrower than those of the commercial sensors CT1050L and ADXL1002 under the current prototype configuration. In addition, although the PMMA tube provides a lateral constraint to reduce off-axis motion, systematic cross-axis sensitivity measurements are not included in the current prototype evaluation and should be further investigated in future work. In addition, unlike commercial sensors with stable sensitivity in the working frequency range, graphene devices show clear frequency-dependent sensitivities. At the same time, the durability test indicates that, before reaching a stable working condition, the graphene aerogel sensor shows an initial decrease in performance. This initial decrease is mainly attributed to slight irreversible deformation and interlayer sliding of graphene sheets within the aerogel structure, together with the gradual release of residual stress during early cyclic compression. To further improve the graphene aerogel sensor for practical applications, optimization of the sensor structure, encapsulation, and conditioning circuit is needed. Finally, although the equipotential clamping structure can reduce the short-circuit risk caused by insulation failure in conventional insulated installation schemes, its mechanical strength, vibration resistance, anti-corrosion packaging, live-line installation procedure, and adaptability to conductors with different specifications still need to be further optimized and verified for practical engineering deployment.

## 6. Conclusions

This study presents a duty-cycled self-powered sensing system for transmission-line vibration monitoring, based on a spatial magnetic field energy harvester, a duty-cycled circuit module, a graphene-based vibration sensor, and a wireless communication unit. The results verify the feasibility of the proposed system from three aspects: spatial magnetic field energy harvesting, closed-loop self-powered operation with LoRa wireless transmission, and low-frequency vibration sensing. These findings indicate the potential of the proposed system for future transmission-line vibration sensing applications.

## Figures and Tables

**Table 1:** Geometric and coil parameters of the magnetic field energy harvester.

Component	Parameter	Value	Unit
Overall Harvester	Dimensions (L × W × H)	60 × 35 × 19.5	mm
Cantilever Beam	Dimensions (L × W × H)	24 × 10 × 0.2	mm
Circular Magnet	Diameter	10	mm
	Thickness	7	mm
Square Magnet	Side Length	10	mm
	Thickness	1	mm
Coil	Inner Diameter	15	mm
	Outer Diameter	29	mm
	Height	5	mm
	Wire Diameter	0.16	mm
	Number of Turns	865	turns
Assembly	Vertical Gap (Beam to Coil)	9.5	mm

**Table 2:** Comparison of the proposed self-powered vibration sensing system with existing self-powered sensing and energy-harvesting approaches.

Ref.	Energy Source	Harvester Mechanism	Power Characteristics	Operating Condition	Sensing Element	Deployment Configuration
[4]	Galloping kinetic energy	Piezoelectric cantilever	155.58 μW (@ 1.3 Hz)	Low-frequency galloping excitation	Piezoelectric ceramic	Insulated suspension
[19]	Aeolian kinetic energy	Triboelectric + EMG	4.2 mW (peak)	Aeolian vibration condition	TENG contact-separation layer	Insulated suspension
[20]	Kinetic energy	Gear-driven DC generator	4.2 W (@ 0.48 m/s)	Wind-driven mechanical excitation	External commercial sensors	Tower-independent structure
[21]	Aeolian kinetic energy	ME-TENG + double EMG	Not reported	Aeolian vibration condition	ME-TENG elastic layer/EMG	Insulated suspension
[22]	Line-current-induced AC magnetic field	Linear PM synchronous generator	160 μW (@ 50 A)	Line current: 50 A	N/A	Proximity deployment
[23]	Line-current-induced AC magnetic field	CT-based inductive energy harvesting	Power consumption: 0.84 mW	Minimum line current: 1 A; operating range: 1–100 A	CT current-sensing circuit	Line-clamped CT
This work	Line-current-induced AC magnetic field	Magnetically driven cantilever with EM induction	729 μW maximum at 0.11 mT	Equivalent starting line current: 28 A	Graphene aerogel	Equipotential clamping

**Table 3:** Comparison of representative commercial and research-grade vibration sensors.

Sensor	Type	Reported Sensitivity	Frequency Range	Measurement Range	Stability/Robustness Specification
CT1050L	Commercial piezoelectric accelerometer	500 mV/g	0.2 Hz–3 kHz	±10 g	Overload shock: 100 g; operating temperature: −20–100 °C
ADXL1002	Commercial MEMS accelerometer	40 mV/g	DC–11 kHz	±50 g	Sensitivity change over temperature: ±5% from −40 to 125 °C; shock survivability: 10,000 g
[26]	Suspended double-layer graphene accelerometer	3.01 mV/g at 160 Hz	Tested at 160 Hz	0.05–1 g tested	Not reported
[14]	Graphene aerogel vibration sensor	Peak sensitivity: ~600 mV/g, frequency dependent	2 Hz–10 kHz	0.5–3.5 g tested	Stable over 12,000 cycles
This work	Graphene aerogel vibration sensor	Peak sensitivity: 625 mV/g, frequency dependent	4 Hz–500 Hz tested	0.7–1.5 g tested	17.64 million cycles; initial decrease followed by stabilization after 8.64 million cycles

## Data Availability

The data presented in this study are contained within the article.

## References

[1] Swain A., Abdellatif E., Mousa A., Pong P.W.T. (2022). Sensor Technologies for Transmission and Distribution Systems: A Review of the Latest Developments. Energies.

[2] Wang L., Li H., Xu L., Li X., Zhang J., Wang X., Chen C. (2022). Design of Intelligent Monitoring System in Galloping Power Transmission Line. Sensors.

[3] Yan Q., Zhou C., Feng X., Deng C., Hu W., Xu Y. (2022). Galloping Vibration Monitoring of Overhead Transmission Lines by Chirped FBG Array. Photonic Sens..

[4] Gao S., Zeng X., Tao B., Ke T., Feng S., Chen Y., Zhou J., Lan W. (2023). Self-Powered Sensing of Power Transmission Lines Galloping Based on Piezoelectric Energy Harvesting. Int. J. Electr. Power Energy Syst..

[5] Riba J.-R., Moreno-Eguilaz M., Bogarra S. (2022). Energy Harvesting Methods for Transmission Lines. Appl. Sci..

[6] Yang F., Du L., Chen W., Li J., Wang Y., Wang D. (2017). Hybrid Energy Harvesting for Condition Monitoring Sensors in Power Grids. Energy.

[7] CIGRE Task Force B2.11.06 (2007). State of the Art of Conductor Galloping.

[8] Gurung C.B., Yamaguchi H., Yukino T. (2003). Identification and Characterization of Galloping of Tsuruga Test Line Based on Multi-Channel Modal Analysis of Field Data. J. Wind. Eng. Ind. Aerodyn..

[9] Zanelli F., Mauri M., Castelli-Dezza F., Tarsitano D., Manenti A., Diana G. (2022). Analysis of Wind-Induced Vibrations on HVTL Conductors Using Wireless Sensors. Sensors.

[10] Huang X., Zhao L., Chen G. (2016). Design of a Wireless Sensor Module for Monitoring Conductor Galloping of Transmission Lines. Sensors.

[11] Gao K., Zhang Z., Weng S., Zhu H., Yu H., Peng T. (2022). Review of Flexible Piezoresistive Strain Sensors in Civil Structural Health Monitoring. Appl. Sci..

[12] Irani F.S., Shafaghi A.H., Tasdelen M.C., Delipinar T., Kaya C.E., Yapici G.G., Yapici M.K. (2022). Graphene as a Piezoresistive Material in Strain Sensing Applications. Micromachines.

[13] Kim K., Kim J., Jiang X., Kim T. (2021). Static Force Measurement Using Piezoelectric Sensors. J. Sens..

[14] Wang Z., Xiao Z., Mei J., Wang Y., Zhang X., Wei X., Liu H., Xie S., Zhou W. (2023). Graphene Aerogel-Based Vibration Sensor with High Sensitivity and Wide Frequency Response Range. Nano Res..

[15] Rayleigh J.W. (1894). The Theory of Sound.

[16] Qiu L., Liu J.Z., Chang S.L.Y., Wu Y., Li D. (2012). Biomimetic superelastic graphene-based cellular monoliths. Nat. Commun..

[17] Cheng S., Wang N., Arnold D.P. (2007). Modeling of magnetic vibrational energy harvesters using equivalent circuit representations. J. Micromech. Microeng..

[18] Halim M.A., Rendon-Hernandez A.A., Smith S.E., Samman J.M., Garraud N., Arnold D.P. (2021). Miniature Electrodynamic Wireless Power Transmission Receiver Using a Micromachined Silicon Suspension. J. Microelectromech. Syst..

[19] Wei B., Peng L., Li S., Huang S. (2026). Self-Powered Vibration Sensor and Monitoring System for Overhead Power Transmission Lines. IEEE Sens. J..

[20] Tan Y., Li S., Zhang W., Zhou Y., He Y., Ren L. (2023). Converting energy from overhead transmission line vibrations using a low-frequency and low-amplitude harvester in a smart grid. Front. Energy Res..

[21] Gao S., Feng S., Wang J., Wu H., Chen Y., Zhang J., Li Y., Wang R., Luo X., Wei H. (2023). Hybridized Triboelectric-Electromagnetic Aeolian Vibration Generator as a Self-Powered System for Efficient Vibration Energy Harvesting and Vibration Online Monitoring of Transmission Lines. ACS Appl. Mater. Interfaces.

[22] Hosseinimehr T., Tabesh A. (2016). Magnetic Field Energy Harvesting from AC Lines for Powering Wireless Sensor Nodes in Smart Grids. IEEE Trans. Ind. Electron..

[23] Chen H., Qian Z., Liu C., Wu J., Li W., He X. (2021). Time-Multiplexed Self-Powered Wireless Current Sensor for Power Transmission Lines. Energies.

[24] Wang Z., Zhao Z., Tu Q., Yao C., Liu Z., Zhou C., Xu L., Guo S., Meng C., Shao G. (2026). Graphene Aerogel-Based Flexible Pressure Sensor for Physiological Signal Detection and Human–Machine Interaction. Nano-Micro Lett..

[25] Li Y., Dou W., Zhou C., Wang X., Yang A., Zhang Y., Qiao D. (2021). A Microtester for Measuring the Reliability of Microdevices in Controlled Environmental Conditions. Micromachines.

[26] Ding J., Ma H., He C., Zhang W., Fan X. (2025). Two Layers of Carbon Atoms Enable Ultrasensitive Detection of Acceleration. ACS Nano.

